# Circle Fitting Based Image Segmentation and Multi-Scale Block Local Binary Pattern Based Distinction of Ring Rot and Anthracnose on Apple Fruits

**DOI:** 10.3389/fpls.2022.884891

**Published:** 2022-06-09

**Authors:** Qin Feng, Shutong Wang, He Wang, Zhilin Qin, Haiguang Wang

**Affiliations:** ^1^College of Plant Protection, China Agricultural University, Beijing, China; ^2^College of Plant Protection, Hebei Agricultural University, Baoding, China; ^3^Forest Pest Management and Quarantine Station of Beijing, Beijing, China

**Keywords:** apple ring rot, apple anthracnose, image distinction, circle fitting, multi-scale block local binary pattern, support vector machine, random forest

## Abstract

Ring rot caused by *Botryosphaeria dothidea* and anthracnose caused by *Colletotrichum gloeosporioides* are two important apple fruit diseases. It is critical to conduct timely and accurate distinction and diagnosis of the two diseases for apple disease management and apple quality control. The automatic distinction between the two diseases was investigated based on image processing technology in this study. The acquired disease images were preprocessed via image scaling, color image contrast stretching, and morphological opening and closing reconstruction. Then, two lesion segmentation methods based on circle fitting were proposed and used to conduct lesion segmentation. After comparison with the manual segmentation results obtained via the software Adobe Photoshop CC, Lesion segmentation method 1 was chosen for further disease image processing. The gray images on the nine components in the RGB, HSI, and L*a*b* color spaces of the segmented lesion images were filtered by using multi-scale block local binary pattern operators with the sizes of pixel blocks of 1 × 1, 2 × 2, and 3 × 3, respectively, and the corresponding local binary pattern (LBP) histogram vectors were calculated as the features of the lesion images. Subsequently, support vector machine (SVM) models and random forest models were built based on individual LBP histogram features or different LBP histogram feature combinations for distinguishing the diseases. The optimal SVM model with the distinction accuracies of the training and testing sets equal to 100 and 95.12% and the optimal random forest model with the distinction accuracies of the training and testing sets equal to 100 and 90.24% were achieved. The results indicated that the distinction between the two diseases could be implemented with high accuracy by using the proposed method. In this study, a method based on image processing technology was provided for the distinction of ring rot and anthracnose on apple fruits.

## Introduction

Apple is a kind of fruit with great commercial value, and it is an important kind of export fruit in China (Chen et al., 2010). Apple ring rot caused by *Botryosphaeria dothidea* and apple anthracnose caused by *Colletotrichum gloeosporioides* is two common diseases on apple fruits (Li B. H. et al., 2013; Hu et al., 2016). These two kinds of diseases form lesions on the apple fruit surface and cause decay on apple fruits, resulting in severe yield losses and quality declines of apple fruits. Lesions on apple fruits caused by ring rot are usually very similar to those caused by anthracnose. Agricultural technicians with rich practical experience are required to differentiate and identify the two apple fruit diseases accurately. The conventional diagnosis method of the diseases mainly relies on naked-eye symptom observations conducted by experienced agricultural technicians. This method is time-consuming and laboursome. In addition, there are not enough agricultural technicians to meet the actual needs of apple production. Therefore, it is necessary to explore a rapid, accurate, convenient, and highly automated disease identification method.

Image processing technology has been widely applied in the diagnosis, identification, and monitoring of plant diseases (Sankaran et al., 2010; Barbedo, 2016; Vishnoi et al., 2021), such as wheat diseases (Li et al., 2012; Johannes et al., 2017; Deng et al., 2021), maize diseases (DeChant et al., 2017; Chen et al., 2021), rice diseases (Phadikar et al., 2013; Lu et al., 2017; Narmadha et al., 2022), cotton diseases (Camargo and Smith, 2009; Caldeira et al., 2021), soybean diseases (Pires et al., 2016; Shrivastava et al., 2017; Araujo and Peixoto, 2019), cucumber diseases (Vakilian and Massah, 2013; Zhang S. W. et al., 2017; Kainat et al., 2021), tomato diseases (Yamamoto et al., 2017; Trivedi et al., 2021), grape diseases (Tian et al., 2007; Oberti et al., 2014; Zhu et al., 2020), and citrus diseases (Pydipati et al., 2006; Sankaran et al., 2013). Moreover, image processing technology has been used to make disease severity assessments (Li et al., 2011; Barbedo, 2014; Vieira et al., 2014; Shrivastava et al., 2015; Ganthaler et al., 2018), conduct pathogen identification (Chesmore et al., 2003; Deng et al., 2012; Wang et al., 2021), and perform automatic counting of pathogen spores (Li X. L. et al., 2013; Li et al., 2017). It is convenient and rapid to perform plant disease identification using image processing technology, and automatic disease identification can be realized, indicating that the image-based plant disease identification method has a good application prospect. However, most of the reported related studies focused on the diagnosis and identification of plant leaf diseases.

There have been some reports on image-based distinction and recognition of apple diseases (Yin et al., 2012; Huo et al., 2013; Dubey and Jalal, 2014, 2016; Omrani et al., 2014; Tan et al., 2015; Wang et al., 2015; Zhang C. L. et al., 2017; Liu et al., 2018; Bansal et al., 2021; Ortega-Sánchez et al., 2022), but few of them focused on the distinction and recognition of apple fruit diseases. The two related studies conducted by Yin et al. (2012) and Huo et al. (2013), respectively, were based on 78 low-resolution images of three kinds of apple fruit diseases including apple ring rot, apple anthracnose, and new apple ring rot (26 images per apple fruit disease) that were taken by using mobile phone in natural scenes, an improved level set interactive segmentation method was used to perform segmentation operation on the preprocessed images, and then six color features, eight texture features, and seven shape features were extracted. Based on the 15 texture and shape features, Yin et al. (2012) developed a support vector machine (SVM) model with a linear kernel function to identify the three kinds of apple fruit diseases, and average identification accuracy of 90.00% was achieved. Based on the eight texture features extracted from the segmented disease images, Huo et al. (2013) built the identification models of the three kinds of apple fruit diseases using three methods including gray relation analysis, SVM, and compressive sensing, the average identification accuracies of 86.67, 90, and 90%, respectively, were obtained for the three models, respectively. Tan et al. (2015) used a deep learning neural network based on flexible momentum to identify the images of diseased apple fruits and achieved a recall rate of 98.4%. Wang et al. (2015) developed a convolutional neural network (CNN) based on a variable impulse learning algorithm to conduct the identification of 100 images of diseased apple fruits, and the identification accuracy was 97.45%. The overall accuracy of 91.1% was obtained by Nachtigall et al. (2017) using CNN to identify healthy apple fruits and unhealthy apple fruits in five disorders including scab caused by *Venturia inaequalis*, alternaria rot caused by *Alternaria alternata*, bull’s eye rot caused by *Cryptosporiopsis perennans*, penicillium rot caused by *Penicillium expansum*, and bitter pit (calcium deficiency) based on the images acquired under controlled conditions.

The quality of plant disease images acquired in natural scenes is usually affected by many factors such as uneven illumination, complex background, and blurred edges. It is necessary to explore an accurate and highly automated image segmentation method to segment these disease images. Furthermore, feature extraction after image segmentation is particularly important for image recognition. The local binary pattern (LBP) operator is a local texture descriptor (Ojala et al., 1996). Because of its characteristics of gray-scale invariance, simple calculation, and insensitivity to illumination changes, this operator is widely used in the fields such as medical image recognition (Nanni et al., 2012; Panda et al., 2018) and face recognition (Ahonen et al., 2006; Yang and Chen, 2013; Lu et al., 2018). The LBP operator has also been applied in the image-based recognition of plant diseases. Leiva-Valenzuela and Aguilera (2013) implemented image-based detection of fungally decayed, shriveled, and mechanically damaged blueberries based on the 951 extracted features including LBP features. In a study conducted by Dubey and Jalal (2014), based on apple fruit images of apple blotch, apple rot, apple scab, and normal apple, the *K*-means clustering technique was applied to perform image segmentation, color and texture features including global color histogram, color coherence vector, color difference histogram, structure element histogram, local ternary pattern, completed local binary pattern (CLBP), and LBP was extracted, and then the color, texture, and fused features were applied to identify apple fruit images by using a multi-class support vector machine (MSVM), finally, an average identification accuracy of approximately 90% was obtained. In another study conducted by Dubey and Jalal (2016), the MSVM method was used to classify apple blotch, apple rot, and apple scab based on the color (global color histogram and color coherence vector), texture (LBP and CLBP), and shape (Zernike moments) feature extracted from apple fruit images, and the results showed that classification performance with accuracy more than 90% could be achieved by using the MSVM models built based on CLBP or each feature combination containing color and texture features. Multi-scale block local binary pattern (MB-LBP), a modified LBP operator, can extract texture information at different scales of an image and is not easily affected by image noise (Liao et al., 2007; Zhang et al., 2007). It has been applied in studies on object detection and recognition (Halidou et al., 2014; Li et al., 2015; Kang et al., 2017; Karanwal, 2021). To the best of our knowledge, there are no reports on the distinction between apple ring rot and apple anthracnose by using the image processing method based on LBP features.

In this study, after preprocessing the digital images of apple fruits infected with ring rot and anthracnose acquired in natural scenes, two lesion segmentation methods based on circle fitting were developed and applied to implement lesion segmentation of the disease images. Subsequently, the gray images on the nine components in the RGB, HSI, and L*a*b* color spaces of the segmented lesion images were filtered by using MB-LBP with pixel blocks in different sizes, and the corresponding LBP histogram features were extracted. Finally, based on these features, SVM models and random forest models were developed to distinguish the two kinds of apple fruit diseases. The aim of this study was to provide a rapid and accurate method for the non-destructive distinction of the two diseases in apple fruits.

## Materials and Methods

**FIGURE 1:**
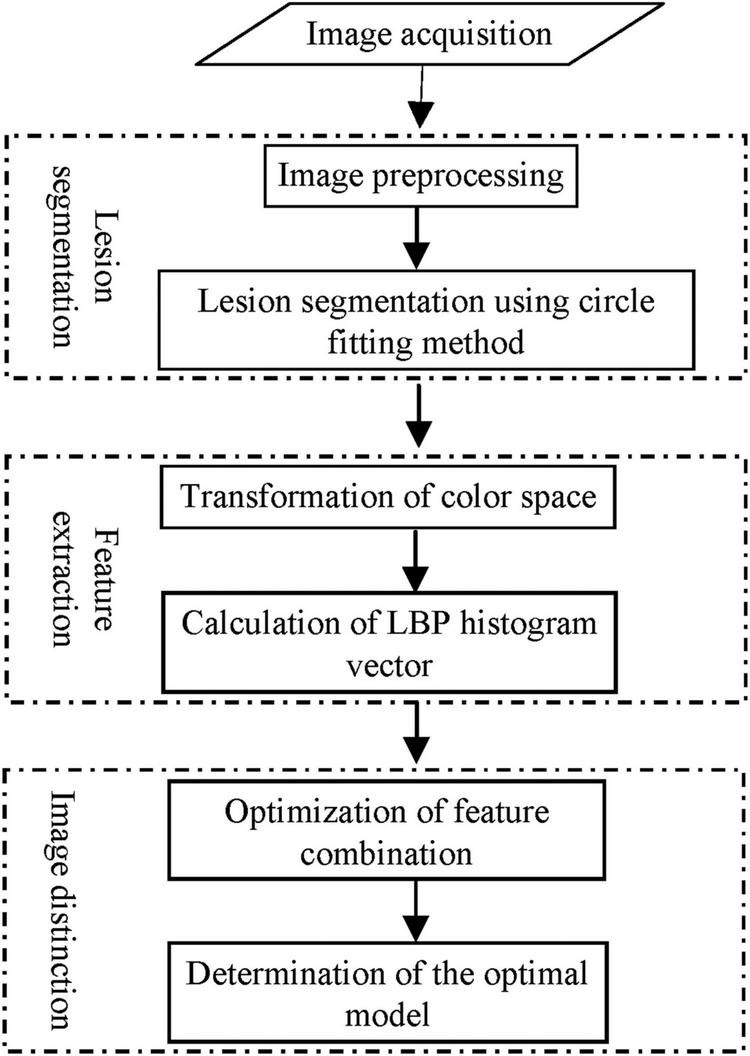
Flow chart of image-based distinction of ring rot and anthracnose on apple fruits.

The distinction of ring rot and anthracnose on apple fruits based on image processing was conducted in accordance with the procedures as shown in Figure 1.

### Acquisition of Disease Images

A total of 123 apple fruit disease images were acquired using different digital cameras under field conditions, including 60 images of apple ring rot and 63 images of apple anthracnose. Most of the disease images used in this study were acquired in the apple orchards in Shangzhuang Experimental Station of China Agricultural University and Sujiatuo County, Haidian District, Beijing, China in the autumn of 2014 by using two digital cameras Canon PowerShot SX100 IS (Canon Inc., Tokyo, Japan) and Canon EOS 700D (Canon Inc., Tokyo, Japan), and were acquired in the apple orchards in Sujiatuo County, Haidian District, Beijing, China in the autumn of 2015 by using the digital camera Canon PowerShot SX100 IS. The other disease images were provided by Shutong Wang from the College of Plant Protection, Hebei Agricultural University, Baoding, China, and He Wang from Forest Pest Management and Quarantine Station of Beijing, Beijing, China.

### Image Preprocessing

Preprocessing operations of the acquired disease images, including image scaling, color image contrast stretching, and morphological opening and closing reconstruction were performed by using the image processing toolbox in the software MATLAB R2013b (MathWorks, Natick, MA, United States).

#### Image Scaling

Because the disease images were acquired by using different digital cameras with different settings, there were obvious differences among the images in size. In order to process the disease images by using the same morphological operations, it is necessary to resize them into the same size range. In this study, the acquired disease images were scaled with an equal ratio in the range of 1,000 × 1,000 pixels to 2,000 × 2,000 pixels.

#### Color Image Contrast Stretching

Color image contrast stretching is conducive to the enhancement of the color difference between the lesions and the surrounding background, and this operation facilitates the subsequent lesion image segmentation. Color image contrast stretching was operated by using the following MATLAB code: *rgbstr* = imadjust(*rgb*, stretchlim(*rgb*)), where *rgb* is the RGB color image to be processed, and *rgbstr* is the processed image.

#### Morphological Opening and Closing by Reconstruction Operations

Morphological opening and closing by reconstruction operations can reduce the noise interference to the real edges (Wang et al., 2008; Zhang and Wang, 2009). In this study, the morphological opening and closing by reconstruction operations of *R, G*, and *B* color components were performed using the circular structure element (disk) with a radius of 10, and then the obtained three color component images were integrated into a new color image by using the function “cat” in the MATLAB R2013b software.

### Lesion Image Segmentation

The backgrounds of apple disease images obtained in natural scenes are usually complex, mainly including soil, branches, and leaves of apple trees, other green plants, and local strong reflection. Automatic and accurate segmentation of lesions on the surface of apple fruit from the complex backgrounds is crucial for disease distinction and identification. Generally, the lesions of ring rot and anthracnose on apple fruits have two distinct characteristics. Firstly, the lesions are usually brown, thus the red component of the lesions in a disease image is often greater than the green component. This characteristic can be used to distinguish green elements such as the leaves of apple trees and other green plants in the background. Secondly, the lesions are located on the surface of apple fruit and are usually nearly round, and this characteristic can be used to distinguish branches of apple trees, soil, and other backgrounds with similar colors to the lesions. In this study, according to the above characteristics, the approximate position of apple fruits in a disease image was determined firstly, and then lesion segmentation was conducted by using the lesion image segmentation methods based on circle fitting. The steps for lesion image segmentation in detail are as follows.

**Step 1.** To make full use of the image color information, the gradient of the integrated color image obtained after morphological opening and closing by reconstruction operations was first calculated. Assuming that *c*(*x, y*) is the gradient of any point (*x, y*) in the color image, it can be calculated according to the method described by Gonzalez and Woods (2011), which can be expressed as follows.

Let *r, g*, and *b* be the unit vectors of the *R*-axis, *G*-axis, and *B*-axis in the RGB color space, respectively, and the vectors *u* and *v* can be defined as:


(1) $$u=\frac{\partial R}{\partial x}r+\frac{\partial G}{\partial x}g+\frac{\partial B}{\partial x}b$$

and


(2) $$v=\frac{\partial R}{\partial y}r+\frac{\partial G}{\partial y}g+\frac{\partial B}{\partial y}b$$

Let *g*_*xx*_, *g*_*yy*_, and *g*_*xy*_ represent the dot products of these vectors *u* and *v*, as follows:


(3) $$g_{xx}=u\cdot u=u^{T}u={\left|\frac{\partial R}{\partial x}\right|}^{2}+{\left|\frac{\partial G}{\partial x}\right|}^{2}+{\left|\frac{\partial B}{\partial x}\right|}^{2}$$

(4) $$g_{yy}=v\cdot v=v^{T}v={\left|\frac{\partial R}{\partial y}\right|}^{2}+{\left|\frac{\partial G}{\partial y}\right|}^{2}+{\left|\frac{\partial B}{\partial y}\right|}^{2}$$

and


(5) $$g_{xv}=u\cdot v=u^{T}v=\frac{\partial R}{\partial x}\frac{\partial R}{\partial y}+\frac{\partial G}{\partial x}\frac{\partial G}{\partial y}+\frac{\partial B}{\partial x}\frac{\partial B}{\partial y}$$

here, the direction of the maximum change rate of *c*(*x, y*) can be given by the angle θ(*x*, *y*), which can be calculated by using the following formula:


(6) $$\theta \left(x,y\right)=\frac{1}{2}\text{arctan}\left[\frac{2g_{xy}}{g_{xx}-g_{yy}}\right]$$

and the value of the change rate at point (*x, y*) in the direction of the angle θ(*x*, *y*) can be given by using the following formula:


(7) $$F_{\theta }\left(x,y\right)=\{\frac{1}{2}[\left(g_{xx}+g_{xy}\right)+\left(g_{xx}-g_{xy}\right)\text{cos}2\theta \left(x,y\right)+2g_{xy}\text{sin}2\theta \left(x,y\right)]\}{}^{\frac{1}{2}}$$

The partial derivatives of Formulas (3), (4), and (5) can be calculated by using the Sobel operator, and then the gradient of any point (*x, y*) can be calculated.

**Step 2.** Edge detection of the gradient image generated in Step 1 was carried out by using the Canny operator. For the Canny operator, the default values were used for the sensitivity thresholds, and the standard deviation of the Gaussian filter, σ(sigma), was set to 20. The purpose of edge detection is to preserve the real edge, remove the false edge, and present the edge image in a binary pattern. In the binary edge image, the edge of the junction of the diseased and healthy regions may have breakpoints. Therefore, in this study, the circular structure element with a radius of 2 was used to conduct a dilation operation on the edge image to obtain the continuous lesion edge as much as possible.

**Step 3.** To remove the green background such as the leaves of the apple tree and other green plants from the image, the pixel points with the green component greater than the red component were assigned a value of 0, and the other pixel points were assigned a value of 1, and then the green-background-subtracted binary image can be obtained. The green apple fruits in the image can also be removed in the process of the background subtraction. Because the lesions of the two apple fruit diseases are usually brown for which the green component is much smaller than the red component, they could still be completely retained in the image after the background subtraction, and thus the subsequent lesion extraction will not be affected. The binary edge image obtained in Step 2 was inversed, and then multiplied with the green-background-subtracted binary image, thus a new binary image was obtained.

**Step 4.** Cavity filling of the binary image obtained in Step 3 was carried out. To avoid the adhesion between apple fruits and the background in the image and remove the relatively small background target, the circular structure element with a radius of 50 was used to conduct the opening operation on the binary image obtained after cavity filling.

**Step 5.** The areas of all the remaining connected components were calculated, and any region for which the area of the connected component was less than two-thirds of the area of the maximum connected component was removed. The retained regions were regarded as the regions where apple fruits may exist.

**Step 6.** Assuming that the number of the retained regions was *M*, that is, the number of the regions where apple fruits may exist was *M*, let *j* = 1, then the convex hull of the *j*th apple fruit region was calculated and the region contour was extracted. Considering that apple fruits are usually near-circular, circle fitting of the *j*th apple fruit region was carried out based on the contour combined with the least square method. Circular curve fitting by using the least square method was carried out according to the specific calculation method as described by Li and He (2013), which can be listed as follows.

Suppose the formula of the circular curve to be fitted is:


(8) $$Rad^{2}={\left(x-Ax\right)}^{2}+{\left(y-By\right)}^{2}$$

in which *Rad* is the radius of the circle curve, and *Ax* and *By* are the abscissa and ordinate of the circle center, respectively.

Suppose the pixel point set on the contour line is (*x*_*i*_, *y*_*i*_) where *i* = 1, 2, …, *N*, and *N* represent the number of pixel points on the contour line. The distance from the *i*th pixel point in the set to the circle center is *d*_*i*_, then


(9) $$d_{i}^{2}={\left(x_{i}-Ax\right)}^{2}+{\left(y_{i}-By\right)}^{2}$$

The difference between the square of the distance from the point (*x*_*i*_, *y*_*i*_) to the circle center and the square of the radius of the circular curve, δ_*i*_, can be described as the following formula:


(10) $$\delta_{i}=d_{i}^{2}-Rad^{2}={\left(x_{i}-Ax\right)}^{2}+{\left(y_{i}-By\right)}^{2}-Rad^{2}=x_{i}^{2}+y_{i}^{2}+ax_{i}+by_{i}+c$$

Let *Q* (*a, b, c*) be the sum of squares of δ_*i*_, then


(11) $$Q\left(a,b,c\right)=\sum \delta_{i}^{2}=\sum {\left[x_{i}^{2}+y_{i}^{2}+ax_{i}+by_{i}+c\right]}^{2}$$

The least-square method was used to calculate and achieve the optimal fitted circular curve, that is, the parameters *a, b*, and *c* were calculated to minimize *Q* (*a, b, c*). The partial derivatives of *Q* (*a, b, c*) with respect to *a, b*, and *c* were calculated, respectively, and then were set to 0, thus the extreme points could be achieved and the values of the corresponding parameters *a, b*, and *c* could be obtained. The partial derivatives of *Q* (*a, b, c*) with respect to *a, b*, and *c* were calculated according to the following Formulas (12), (13), and (14), respectively.


(12) $$\frac{\partial Q\left(a,b,c\right)}{\partial a}=\sum 2\left(x_{i}^{2}+y_{i}^{2}+ax_{i}+by_{i}+c\right)x_{i}=0$$

(13) $$\frac{\partial Q\left(a,b,c\right)}{\partial b}=\sum 2\left(x_{i}^{2}+y_{i}^{2}+ax_{i}+by_{i}+c\right)y_{i}=0$$

(14) $$\frac{\partial Q\left(a,b,c\right)}{\partial c}=\sum 2\left(x_{i}^{2}+y_{i}^{2}+ax_{i}+by_{i}+c\right)=0$$

After the values of the corresponding parameters *a, b*, and *c* were obtained, the fitted values of *Ax, By*, and *Rad* could be estimated according to the following Formulas (15), (16), and (17), respectively.


(15) $$Ax=\frac{a}{-2}$$

(16) $$By=\frac{b}{-2}$$

(17) $$Rad=\frac{1}{2}\sqrt{a^{2}+b^{2}-4c}$$

After circle fitting of the *j*th apple fruit contour was conducted, the distances between all the pixel points in the image and the circle center were calculated, then the pixel points with a distance less than the circle radius were assigned a value of 1 and the other pixel points were assigned a value of 0, and thus the binary image of the *j*th apple fruit region was obtained.

**Step 7.** Two methods were tried to find the real lesion edges in this study, thus the corresponding lesion image segmentation methods were classified as Lesion segmentation method 1 and Lesion segmentation method 2, respectively. For Lesion segmentation method 1, the binary edge image obtained in Step 2 was multiplied with the circle fitting binary image of the *j*th apple fruit region, in order to retain the edge inside the apple fruit region and remove the edge outside the apple fruit region; the convex hull areas of all the edges were calculated, and the edge with the largest convex hull area was considered as the edge of the junction of the diseased and healthy regions; then the convex hull contour corresponding to this edge was calculated, and the circle fitting method of apple fruit region was used to fit the contour, finally the region obtained by circle fitting was treated as the region where the disease lesion was located. For Lesion segmentation method 2, firstly, the circular structure element with a radius of 20 was used to conduct the closing operation on the image obtained by the opening operation in Step 4, and a binary image was obtained. The purpose of this operation was to reduce the possible depressions in the retained regions. Subsequently, the binary edge image obtained in Step 2 was multiplied with the circle fitting binary image of the *j*th apple fruit region and then was multiplied with the above binary image obtained by closing operation, aiming to retain the edge inside the apple fruit region and remove the edge outside the apple fruit region. The remaining procedures of Lesion segmentation method 2 were the same as that of Lesion segmentation method 1.

**Step 8.** Let *j* = *j*+1, if *j* ≤ *M*, then Steps 6 and 7 will be repeated, otherwise the operations for the lesion image segmentation will be finished.

After segmentation, each pixel in a lesion image was determined as a lesion pixel or a healthy pixel. The evaluation of image segmentation performance can be conducted by referring to the evaluation method of a binary classification model (Powers, 2011). The manual segmentation method using the Adobe Photoshop CC software was utilized to conduct segmentation of the lesion images, and the segmentation results were considered as references. The segmentation results obtained by using the manual segmentation method were compared with those obtained by using the two segmentation methods described above, and Recall, Precision, and Score (Qin et al., 2016) were used as the indices to evaluate the above two segmentation methods. The three indices were calculated according to the following formulas as described by Qin et al. (2016):


(18) $$\text{Recall}=\frac{N_{1}}{N_{2}}$$

(19) $$\text{Precision}=\frac{N_{1}}{N_{3}}$$

(20) $$\text{Score}=\frac{\text{Recall}+\text{Precision}}{2}$$

where *N*_1_ is the total number of lesion pixels in a lesion image correctly determined by using one of the segmentation methods described above, *N*_2_ is the total number of lesion pixels in the lesion image determined by using the manual segmentation method, and *N*_3_ is the total number of the pixels in the lesion image. All of these three indices range from 0 to 1. To evaluate the performances of the two lesion segmentation methods, the image dataset of apple ring rot comprising 60 images, the image dataset of apple anthracnose comprising 63 images, and the aggregated image dataset comprising all of the 123 images, were constructed by using the acquired images after preprocessing. The segmentation method with larger values of Recall, Precision, and Score, was chosen as the automatic lesion segmentation method for further disease image processing and disease distinction.

### Extraction of Local Binary Pattern Histogram Features From the Segmented Lesion Images

To reduce the influence of illumination on the image features, MB-LBP operators with pixel blocks in different sizes were used to filter the gray images on the nine components in the RGB, HSI, and L*a*b* color spaces of the segmented lesion images, and then the corresponding LBP histogram vectors were calculated.

**FIGURE 2:**
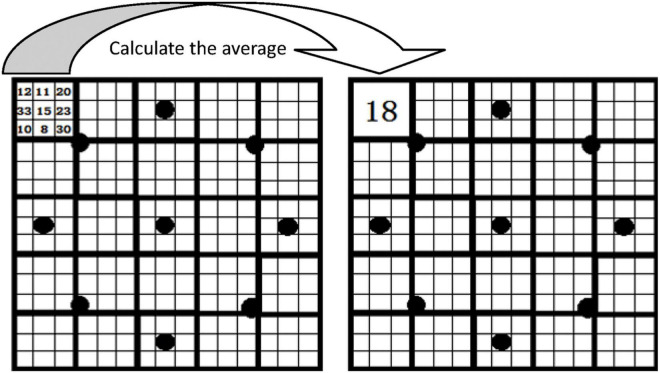
The diagram of the MB_3_-LBP operator.

Initially, all of the segmented lesion images were resized to 256 × 256 pixels. The three-scale sizes of pixel blocks (sub-regions) were set as 1 × 1 pixels, 2 × 2 pixels, and 3 × 3 pixels, and the corresponding MB-LBP operators were recorded as MB_1_-LBP, MB_2_-LBP, and MB_3_-LBP, respectively. For each MB-LBP operator, the number of neighborhoods was set to 8, and the neighborhood radius was set to 2. The MB_3_-LBP operator, as shown in Figure 2, was taken as an example. In Figure 2, each small square surrounded by thin black lines represents a pixel, and each square (sub-region) enclosed by thick black lines represents a pixel block. The gray value of each pixel block is the average of the gray values of nine (3 × 3) pixels included in the corresponding block. The black point at the center of the center block is labeled as the center point, and the eight surrounding black points are labeled as to its eight neighborhood points. For the black point located at the center of a pixel block, the gray value of the pixel block is used directly as the value of this black point. For the black point is not located at the center of a pixel block, the gray value of the pixel block is determined by using the bilinear interpolation method. By comparing the gray value of each neighborhood point with that of the center point, an 8-bit binary number is obtained, which is then used as the response value of the center point. MB_1_-LBP operator and MB_2_-LBP operator are the same as the MB_3_-LBP operator except for the size of each pixel block.

The gray images on the nine components in the RGB, HSI, and L*a*b* color spaces of the segmented lesion images were filtered by using MB_1_-LBP with the size of the pixel block of 1 × 1, MB_2_-LBP with the size of the pixel block of 2 × 2, and MB_3_-LBP with the size of the pixel block of 3 × 3, respectively, and the corresponding local binary pattern histogram vectors were calculated as the features of the lesion images (Zhang et al., 2013). In this study, the uniform LBP operator with 59 histogram bins that include 58 uniform histogram bins and one non-uniform histogram bin, was used for the calculation of the LBP histogram. Finally, the LBP histogram vector in 59 dimensions was obtained. The algorithm in detail was described by Zhang et al. (2007, 2013).

### Disease Distinction Model Building Based on Local Binary Pattern Histogram Features of the Segmented Lesion Images

From all of the acquired apple disease images, 40 images of ring rot and 42 images of anthracnose were randomly selected to form the training set, and the remaining 20 images of ring rot and the remaining 21 images of anthracnose were used to form the testing set. Disease distinction models were built by using two modeling methods including the SVM method and the random forest method.

The LBP histogram features extracted by using the MB-LBP operators are in a large number of dimensions. The SVM method can be applied to effectively solve the data problems of small samples, non-linearity, high dimensions, and local minima (Cortes and Vapnik, 1995; Burges, 1998). Therefore, the SVM method was used to build distinction models of the images of ring rot and anthracnose on apple fruits in this study. Based on the LBP histogram features extracted from the segmented lesion images, the SVM models for the distinction of the two kinds of apple diseases were built by using C-SVM in the LIBSVM package developed by Chang and Lin (2011). To build an SVM model, a radial basis function kernel was selected, and the grid search algorithm was used to search for the optimal penalty parameter *C* and the optimal kernel function parameter *g* in the range of 2^–10^–2^10^ with a searching step of 0.4. Based on the training set, the distinction accuracies at all points within the grid were achieved by running three complete cross-validations. When the highest distinction accuracy was achieved, the corresponding values of *C* and *g* were treated as the optimal parameters and were recorded as *C*_*best*_ and *g*_*best*_, respectively. Then, the SVM model was built by using the parameters *C*_*best*_ and *g*_*best*_. The distinction accuracies of the training set and testing set were calculated and were used to evaluate the model distinction performance.

A random forest, composed of multiple decision trees, can realize prediction by integrating the prediction result of each decision tree (Breiman, 2001). This modeling method can deal well with high-dimensional features, and the running speed of the built model is fast. Therefore, the random forest method was used to build distinction models of the images of apple ring rot and apple anthracnose based on the extracted LBP histogram features in this study. To a certain extent, the distinction performance of a random forest model depends on the number of decision trees constituting the model, so it is necessary to try a variety of values and determine the optimal number of decision trees according to the distinction performances of the built random forest models. In this study, during building the random forest models for disease distinction, the number of decision trees was successively assigned as 10, 20, 30, 40, 50, 60, 70, 80, 90, and 100, and the optimal random forest model was determined according to the distinction accuracies of the training set and testing set and the number of decision trees. For the built random forest models with the same distinction accuracies of the training set and testing set, the one with the least number of decision trees was considered the optimal model. For each decision tree, the arithmetic square root ($\sqrt{N}$ or *N*^1/2^) of the total number (*N*) of LBP histogram features used for modeling was treated as the number of features randomly selected. If $\sqrt{N}$ or *N*^1/2^ was a decimal, the integral number obtained by rounding up the decimal was considered the value of the feature number.

## Results

### Results of Image Preprocessing and Image Segmentation

**FIGURE 3:**
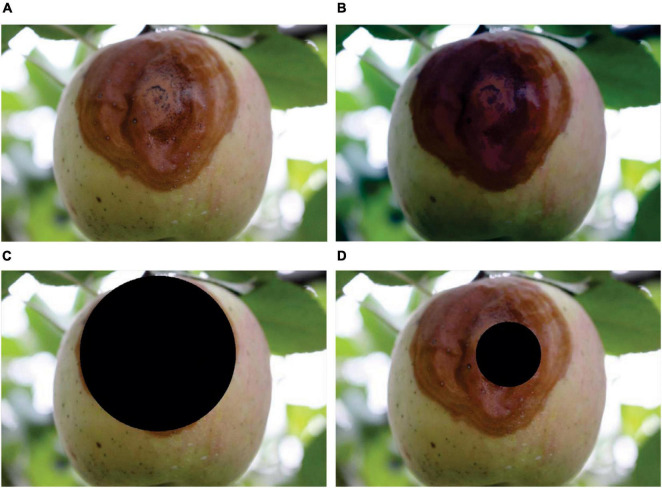
The results of image preprocessing and lesion segmentation for apple ring rot. **(A)** Original color image. **(B)** Image after preprocessing. **(C)** Image after lesion segmentation by using Lesion segmentation method 1 without closing operation. **(D)** Image after lesion segmentation by using Lesion segmentation method 2 with the closing operation. The black areas in panels **(C,D)** are the segmented lesions obtained by using Lesion segmentation method 1 and Lesion segmentation method 2, respectively.

**FIGURE 4:**
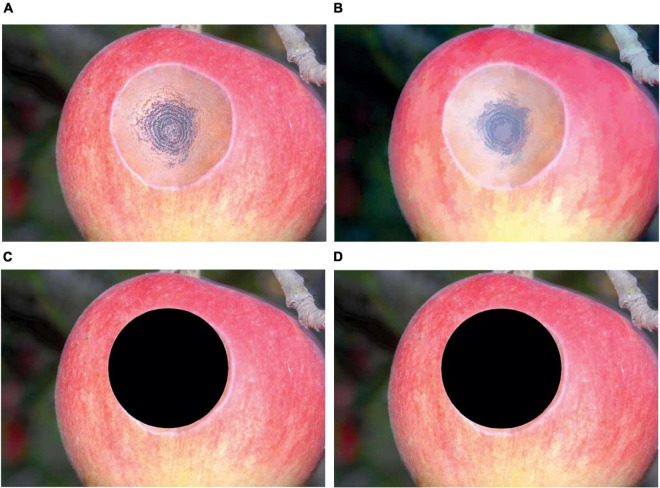
The results of image preprocessing and lesion segmentation for apple anthracnose. **(A)** Original color image. **(B)** Image after preprocessing. **(C)** Image after lesion segmentation by using Lesion segmentation method 1 without closing operation. **(D)** Image after lesion segmentation by using Lesion segmentation method 2 with the closing operation. The black areas in panels **(C,D)** are the segmented lesions obtained by using Lesion segmentation method 1 and Lesion segmentation method 2, respectively.

For apple ring rot, the results of image preprocessing and image segmentation, taking an image as an example, are shown in Figure 3. As shown in Figures 3A,B, after image preprocessing, the color of the lesion region on the surface of the diseased apple was obviously deepened, and the edge of the junction of the diseased and healthy regions became clearer. The results of lesion image segmentation by using Lesion segmentation method 1 and Lesion segmentation method 2 are shown in Figures 3C,D, respectively. The results demonstrated that the size and location of the lesion segmented by using Lesion segmentation method 1 were closer to that of the real lesion than that of the lesion segmented by using Lesion segmentation method 2. For apple anthracnose, there were no relatively obvious differences between the segmentation results of the two segmentation methods, and the satisfactory lesion segmentation performances were achieved by using both Lesion segmentation method 1 and Lesion segmentation method 2. Taking an image of apple anthracnose as an example, the corresponding results of image preprocessing and image segmentation are shown in Figure 4.

After lesion segmentation operations of all the diseased images were conducted by using the two lesion segmentation methods (i.e., Lesion segmentation method 1 and Lesion segmentation method 2), the statistical results of Recalls, Precisions, and Scores for the two methods based on the three image datasets described above are shown in Table 1. The shape of very few lesions was very irregular, e.g., two or more lesions joined together, resulting in the extreme values of Recall, Precision, and Score. To reduce the influence of the extreme values on evaluating the lesion segmentation methods, the mean and median of each evaluation index (Recall, Precision, or Score) were used to evaluate the performances of the two lesion segmentation methods described above.

**TABLE 1:** Statistical comparison of the segmentation effects using the two lesion segmentation methods.

Image dataset	Lesion segmentation method	Recall		Precision		Score	
		Mean	Median	Mean	Median	Mean	Median
Image dataset of apple ring rot	Lesion segmentation method 1	0.93	0.99	0.92	0.95	0.93	0.95
	Lesion segmentation method 2	0.81	0.89	0.93	0.96	0.87	0.93
Image dataset of apple anthracnose	Lesion segmentation method 1	0.95	1.00	0.94	0.97	0.94	0.97
	Lesion segmentation method 2	0.91	0.99	0.96	0.97	0.93	0.97
Aggregated image dataset	Lesion segmentation method 1	0.94	0.99	0.93	0.96	0.93	0.96
	Lesion segmentation method 2	0.86	0.96	0.95	0.97	0.90	0.95

*Aggregated image dataset was obtained after aggregation of the two image datasets of apple ring rot and apple anthracnose.*

For the image dataset of apple ring rot, when Lesion segmentation method 1 was used, the mean and median of the Recalls were 0.93 and 0.99, respectively; the mean and median of the Precisions were 0.92 and 0.95, respectively; and the mean and median of the Scores were 0.93 and 0.95, respectively. For this image dataset, when Lesion segmentation method 2 was used, the mean and median of the Recalls were 0.81 and 0.89, respectively; the mean and median of the Precisions were 0.93 and 0.96, respectively; and the mean and median of the Scores were 0.87 and 0.93, respectively. The results demonstrated that, for the image dataset of apple ring rot, the means and medians of the Recalls and Scores obtained when Lesion segmentation method 1 was used, were higher than those obtained when Lesion segmentation method 2 was used; and the mean and median of the Precisions were similar when Lesion segmentation method 1 and Lesion segmentation method 2 were used, respectively. The results indicated that Lesion segmentation method 1 was more suitable for lesion segmentation of the images of ring rot on apple fruits.

For the image dataset of apple anthracnose, when Lesion segmentation method 1 was used, the mean and median of the Recalls were 0.95 and 1, respectively; the mean and median of the Precisions were 0.94 and 0.97, respectively; and the mean and median of the Scores were 0.94 and 0.97, respectively. When Lesion segmentation method 2 was used on this image dataset, the mean and median of the Recalls were 0.91 and 0.99, respectively; the mean and median of the Precisions were 0.96 and 0.97, respectively; and the mean and median of the Scores were 0.93 and 0.97, respectively. The results demonstrated that, for the image dataset of apple anthracnose, the mean and median of the Recalls, Precisions, or Scores obtained when Lesion segmentation method 1 and Lesion segmentation method 2 were used, respectively, were similar, and all the indices were more than 0.9. The results indicated that these two lesion segmentation methods were both suitable for lesion segmentation of the images of apple anthracnose.

For the image dataset of the aggregated image dataset obtained after aggregation of the two image datasets of apple ring rot and apple anthracnose, when Lesion segmentation method 1 was used, the mean and median of the Recalls were 0.94 and 0.99, respectively; the mean and median of the Precisions were 0.93 and 0.96, respectively; and the mean and median of the Scores were 0.93 and 0.96, respectively. For this aggregated image dataset, when Lesion segmentation method 2 was used, the mean and median of the Recalls were 0.86 and 0.96, respectively; the mean and median of the Precisions were 0.95 and 0.97, respectively; and the mean and median of the Scores were 0.9 and 0.95, respectively. The results showed that, for the aggregated image dataset, the mean and median of the Recalls or Scores obtained when Lesion segmentation method 1 was used, were both higher than those obtained when Lesion segmentation method 2 was used, and the mean and median of the Precisions obtained when the former method was used were similar to those obtained when the latter method was used.

The results described above indicated that Lesion segmentation method 1 was more suitable for lesion segmentation of the images of ring rot and anthracnose on apple fruits. Therefore, Lesion segmentation method 1 was selected for realizing the automatic segmentation of the lesion images of the two apple fruit diseases in this study.

### Distinction Results of the Support Vector Machine Models Based on the Local Binary Pattern Histogram Features

The distinction results of the SVM models based on the LBP histogram features of the gray images on each individual component in the RGB, HSI, and L*a*b* color spaces of the segmented lesion images are shown in Tables 2–4, respectively. R1 denoted the LBP histogram feature of the gray image of the *R* component of the lesion image filtered by the MB_1_-LBP operator, and R2 denoted the LBP histogram feature of the gray image of the *R* component of the lesion image filtered by the MB_2_-LBP operator, R1G1 denoted the combination of R1 and G1, and the rest features’ names could be deduced by analogy. The results showed that the optimal SVM model for disease distinction was built based on the feature L1a1 and that this SVM model had the best distinction performance. For this optimal SVM model, the parameters *C*_*best*_ and *g*_*best*_ were 12.126 and 0.144, respectively, and the distinction accuracy of the training set was 100% and the distinction accuracy of the testing set was 95.12%. The model for which the distinction performance ranked second among all the built SVM models, was built based on the feature R1B1 with the parameters *C*_*best*_ and *g*_*best*_ of 21.112 and 0.047. For this model, the distinction accuracies of the training set and testing set were 96.34% and 92.68%, respectively. The model for which the distinction performance ranked third among all the built SVM models, was built based on the feature R1 with the parameters *C*_*best*_ and *g*_*best*_ of 21.112 and 0.082. For this model, the distinction accuracy of the training set was 93.9% and the distinction accuracy of the testing set was 90.24%. The SVM model was built based on the feature R1G1B1 with the optimal parameters *C*_*best*_ and *g*_*best*_ of 194.012 and 0.016 and the SVM model was built based on the feature L1a1b1 with the optimal parameters *C*_*best*_ and *g*_*best*_ of 6.964 and 0.082, the distinction accuracies of the training set were both 100% and the distinction accuracies of the testing set were both 87.8%. For the SVM model built based on the feature L1b1 with the optimal parameters, *C*_*best*_ and *g*_*best*_ of 2.297 and 0.25, the distinction accuracies of the training set and testing set were 97.56 and 85.37%, respectively. For the SVM model built based on the feature R1G1 with the optimal parameters, *C*_*best*_ and *g*_*best*_ of 2.297 and 0.144, the distinction accuracies of the training set and testing set were 92.68 and 85.37%, respectively. The results demonstrated that accurate distinction of apple ring rot and apple anthracnose can be achieved by using the SVM modeling method based on LBP histogram features. The LBP histogram features used in the above SVM models with satisfactory distinction performances were obtained by filtering the gray images of the related components with the MB_1_-LBP operator. Compared with the other two MB-LBP operators (MB_2_-LBP and MB_2_-LBP), the pixel block of the MB_1_-LBP operator is the smallest, and the highest fineness of the image texture can be obtained after image filtering with it, which may be helpful to improve the ability of the models to distinguish between apple ring rot and apple anthracnose.

**TABLE 2:** Distinction results of the SVM models based on the LBP histogram features of the gray images of the three components in RGB color space of the segmented lesion images.

Feature	The optimal parameters of SVM model		Distinction accuracy of the training set/%	Distinction accuracy of the testing set/%
	*C*_best_	*g*_best_		
R1	21.112	0.082	93.90	90.24
G1	2.297	0.144	80.49	70.73
B1	0.758	0.250	78.05	60.98
R1G1	2.297	0.144	92.68	85.37
R1B1	21.112	0.047	96.34	92.68
G1B1	588.134	0.005	96.34	73.17
R1G1B1	194.012	0.016	100.00	87.80
R2	1.320	2.297	100.00	70.73
G2	2.297	0.435	96.34	58.54
B2	0.758	0.250	75.61	65.85
R2G2	111.430	0.005	95.12	80.49
R2B2	1.320	0.082	87.80	82.93
G2B2	0.758	0.144	78.05	68.29
R2G2B2	1.320	0.082	90.24	80.49
R3	2.297	2.297	100.00	73.17
G3	337.794	0.003	90.24	58.54
B3	21.112	0.027	90.24	68.29
R3G3	2.297	0.250	98.78	75.61
R3B3	21.112	0.027	96.34	82.93
G3B3	64	0.047	100.00	58.54
R3G3B3	4	0.082	97.56	75.61

*R1 represents the LBP histogram feature of the gray image of the R component of the lesion image filtered by the MB_1_-LBP operator; R2 represents the LBP histogram feature of the gray image of the R component of the lesion image filtered by the MB_2_-LBP operator; R1G1 represents the combination of R1 and G1; the rest features’ implication could be deduced by analogy.*

**TABLE 3:** Distinction results of the SVM models based on the LBP histogram features of the gray images of the three components in the HSI color space of the segmented lesion images.

Feature	The optimal parameters of SVM model		Distinction accuracy of the training set/%	Distinction accuracy of the testing set/%
	*C*_*best*_	*g*_*best*_		
H1	0.758	0.082	69.51	68.29
S1	111.430	0.016	82.93	82.93
I1	2.297	0.758	96.34	78.05
H1S1	6.964	0.016	75.61	82.93
H1I1	2.297	0.047	79.27	78.05
S1I1	0.435	0.144	78.05	78.05
H1S1I1	0.435	0.082	75.61	80.49
H2	2.297	0.144	78.05	75.61
S2	337.794	0.005	89.02	46.34
I2	0.758	0.250	79.27	68.29
H2S2	4.000	0.250	100.00	70.73
H2I2	0.758	0.144	81.71	80.49
S2I2	1.320	0.082	81.71	75.61
H2S2I2	0.758	0.250	92.68	80.49
H3	6.964	0.027	75.61	70.73
S3	0.758	0.758	93.90	63.41
I3	21.112	0.002	62.20	65.85
H3S3	1.320	0.144	90.24	75.61
H3I3	1.320	0.435	98.78	82.93
S3I3	1.320	0.758	100.00	70.73
H3S3I3	1.320	0.435	100.00	78.05

*H1 represents the LBP histogram feature of the gray image of the H component of the lesion image filtered by the MB_1_-LBP operator; H2 represents the LBP histogram feature of the gray image of the H component of the lesion image filtered by the MB_2_-LBP operator; H1S1 represents the combination of H1 and S1; the rest features’ implication could be deduced by analogy.*

**TABLE 4:** Distinction results of the SVM models based on the LBP histogram features of the gray images of the three components in the L*a*b* color space of the segmented lesion images.

Feature	The optimal parameters of SVM model		Distinction accuracy of the training set/%	Distinction accuracy of the testing set/%
	*C*_*best*_	*g*_*best*_		
L1	12.126	0.435	100.00	73.17
a1	2.297	1.320	100.00	65.854
b1	1.320	0.758	96.34	80.49
L1a1	12.126	0.144	100.00	95.12
L1b1	2.297	0.250	97.56	85.37
a1b1	2.297	0.758	100.00	70.73
L1a1b1	6.964	0.082	100.00	87.80
L2	1.320	0.758	95.12	63.41
a2	36.758	0.009	80.49	70.73
b2	1.320	0.144	84.15	75.61
L2a2	0.758	0.435	96.34	73.17
L2b2	0.758	0.250	90.24	73.17
a2b2	36.758	0.027	98.78	65.85
L2a2b2	1.320	0.082	95.12	78.05
L3	111.430	0.047	98.78	58.54
a3	337.794	0.009	91.46	56.10
b3	6.964	0.082	91.46	75.61
L3a3	1.320	0.435	100.00	70.73
L3b3	1.320	0.435	100.00	80.49
a3b3	0.758	0.144	87.80	78.05
L3a3b3	1.320	0.144	98.78	80.49

L1 represents the LBP histogram feature of the gray image of the L* component of the lesion image filtered by the MB_1_-LBP operator; L2 represents the LBP histogram feature of the gray image of the L* component of the lesion image filtered by the MB_2_-LBP operator; L1a1 represents the combination of L1 and a1; the rest features’ implication could be deduced by analogy.

### Distinction Results of the Random Forest Models Based on the Local Binary Pattern Histogram Features

The distinction results of the random forest models built based on the LBP histogram features of the gray images of the three components in the RGB, HIS, and L*a*b* color spaces of the segmented lesion images of apple ring rot and apple anthracnose are shown in Tables 5–7, respectively. The results showed that the optimal random forest model for the distinction of the two apple fruit diseases was built with the number of decision trees equal to 30 based on the feature R1B1 and that the distinction performance of this model was the best among all the built random forest models. For this optimal model, the distinction accuracies of the training set and testing set were 100 and 90.24%, respectively. In terms of disease distinction performance, three models tied for second place with the distinction accuracies of the training set and testing set equal to 100 and 87.8%, respectively, among all the built random forest models. Among these three models, one was built with the number of decision trees equal to 90 based on the feature R1, another was built with the number of decision trees equal to 50 based on the feature R1G1B1, and the other was built with the number of decision trees equal to 80 based on the feature L3a3b3. For the random forest model built based on the feature R1G1, H1S1, H1S1I1, I3, H3I3, L1b1, or L3a3 with the number of decision trees corresponding to 60, 60, 90, 80, 100, 70, or 30, the distinction accuracy of the training set was 100% and the distinction accuracy of the testing set was 85.37%. The results demonstrated that accurate distinction of apple ring rot and apple anthracnose can be obtained by using the random forest method based on LBP histogram features.

**TABLE 5:** Distinction results of the random forest models based on the LBP histogram features of the gray images of the three components in RGB color space of the segmented lesion images.

Feature	The number of decision trees built by the best random forest model	Distinction accuracy of the training set/%	Distinction accuracy of the testing set/%
R1	90	100.00	87.80
G1	60	100.00	75.61
B1	100	100.00	75.61
R1G1	60	100.00	85.37
R1B1	30	100.00	90.24
G1B1	80	100.00	80.49
R1G1B1	50	100.00	87.80
R2	90	100.00	78.05
G2	10	98.78	75.61
B2	20	100.00	63.41
R2G2	20	98.78	73.17
R2B2	60	100.00	82.93
G2B2	10	97.56	73.17
R2G2B2	90	100.00	82.93
R3	100	100.00	80.49
G3	70	100.00	73.17
B3	50	100.00	73.17
R3G3	60	100.00	80.49
R3B3	90	100.00	75.61
G3B3	90	100.00	73.17
R3G3B3	50	100.00	75.61

*R1 represents the LBP histogram feature of the gray image of the R component of the lesion image filtered by the MB_1_-LBP operator; R2 represents the LBP histogram feature of the gray image of the R component of the lesion image filtered by the MB_2_-LBP operator; R1G1 represents the combination of R1 and G1; the rest features’ implication could be deduced by analogy.*

**TABLE 6:** Distinction results of the random forest models based on the LBP histogram features of the gray images of the three components in the HSI color space of the segmented lesion images.

Feature	The number of decision trees built by the best random forest model	Distinction accuracy of the training set/%	Distinction accuracy of the testing set/%
H1	90	100.00	70.73
S1	30	100.00	78.05
I1	70	100.00	78.05
H1S1	60	100.00	85.37
H1I1	50	100.00	80.49
S1I1	50	100.00	82.93
H1S1I1	90	100.00	85.37
H2	20	100.00	75.61
S2	40	100.00	82.93
I2	40	100.00	73.17
H2S2	90	100.00	78.05
H2I2	60	100.00	82.93
S2I2	60	100.00	75.61
H2S2I2	90	100.00	80.49
H3	70	100.00	73.17
S3	20	100.00	70.73
I3	80	100.00	85.37
H3S3	60	100.00	80.49
H3I3	100	100.00	85.37
S3I3	50	100.00	80.49
H3S3I3	70	100.00	82.93

*H1 represents the LBP histogram feature of the gray image of the H component of the lesion image filtered by the MB_1_-LBP operator; H2 represents the LBP histogram feature of the gray image of the H component of the lesion image filtered by the MB_2_-LBP operator; H1S1 represents the combination of H1 and S1; the rest features’ implication could be deduced by analogy.*

**TABLE 7:** Distinction results of the random forest models based on the LBP histogram features of the gray images of the three components in the L*a*b* color space of the segmented lesion images.

Feature	The number of decision trees built by the best random forest model	Distinction accuracy of the training set/%	Distinction accuracy of the testing set/%
L1	30	100.00	80.49
a1	60	100.00	75.61
b1	100	100.00	78.05
L1a1	60	100.00	82.93
L1b1	70	100.00	85.37
a1b1	40	100.00	80.49
L1a1b1	60	100.00	82.93
L2	10	97.56	70.73
a2	60	100.00	82.93
b2	80	100.00	78.05
L2a2	40	100.00	80.49
L2b2	30	100.00	80.49
a2b2	50	100.00	80.49
L2a2b2	70	100.00	80.49
L3	60	100.00	73.17
a3	20	100.00	80.49
b3	60	100.00	80.49
L3a3	30	100.00	85.37
L3b3	100	100.00	82.93
a3b3	30	100.00	80.49
L3a3b3	80	100.00	87.80

L1 represents the LBP histogram feature of the gray image of the L* component of the lesion image filtered by the MB_1_-LBP operator; L2 represents the LBP histogram feature of the gray image of the L* component of the lesion image filtered by the MB_2_-LBP operator; L1a1 represents the combination of L1 and a1; the rest features’ implication could be deduced by analogy.

## Discussion and Conclusion

Diseases play important roles in the reduction of the yield and quality of apple fruits. Accurate disease diagnosis is a key prerequisite for the prevention and control of apple diseases. In this study, taking ring rot and anthracnose on apple fruits as the research objects, the lesion image segmentation of the two apple diseases was carried out, and then the MB-LBP features were extracted from the segmented lesion images, finally, the distinction of the images of the two apple diseases was conducted by using both the SVM method and the random forest method. According to the characteristics of the lesions of the two apple diseases, two lesion segmentation methods based on circle fitting, Lesion segmentation method 1 and Lesion segmentation method 2, were developed and compared. The statistical results of three evaluation indices including Recall, Precision, and Score of the two lesion segmentation methods indicated that Lesion segmentation method 1 was better than Lesion segmentation method 2. Therefore, Lesion segmentation method 1 was selected to realize automatic segmentation of the lesion images in this study. To reduce the influence of illumination on the features of lesion images, the gray images of the nine components in the RGB, HSI, and L*a*b* color spaces of the segmented lesion images of the two apple diseases were filtered by using MB_1_-LBP, MB_2_-LBP, and MB_3_-LBP operators, respectively, and the corresponding LBP histogram features were extracted for further disease distinction. The results demonstrated that for the built disease distinction SVM model based on the feature R1, R1G1, R1B1, L1a1, L1b1, R1G1B1, or L1a1b1, the distinction accuracies of the training set and testing set were high, and the satisfactory disease distinction performance was achieved. Among these SVM models, the distinction performance of the model built based on the feature L1a1 was optimal. The obtained results showed that the satisfactory disease distinction performance could be achieved when the random forest model was built based on the features R1, I3, R1G1, R1B1, H1S1, H3I3, L1b1, L3a3, R1G1B1, H1S1I1, or L3a3b3. Among these random forest model models, the distinction performance of the model built based on the feature R1B1 was optimal. For both the SVM model and the random forest model based on the feature R1B1, the distinction accuracies of the training set and testing set were more than 90%, indicating that the feature R1B1 can be utilized to well distinguish between ring rot and anthracnose on apple fruits. In practical applications, the disease image database as a training set may be very large. Generally, the random forest model runs faster than the SVM model, and it is easy to realize large-scale parallel computing and rapid analysis of massive data by using the random forest method. Considering these factors, it is suggested that using R1B1 features to build a random forest model can be carried out for the distinction of the images of ring rot and anthracnose on apple fruits. The results indicated that it is feasible to distinguish between ring rot and anthracnose with typical symptoms on apple fruits by using the method proposed in this study.

Disease images obtained in natural scenes often have complex backgrounds, which can induce uneven illumination, various noises, blurred lesion edges, and other phenomena in the images. Therefore, under these circumstances, it is difficult to conduct complete lesion image segmentation. Zou et al. (2010) used multi-threshold methods to segment apple images from the black background, and then used a flooding algorithm and a snake algorithm to conduct the detection of apple fruit defects, thus the in-line detection of apple quality was realized. The lesion image segmentation method of the two apple fruit diseases based on circle fitting proposed in this study can realize the automatic segmentation of lesion images without human interaction. Even if the image background, such as a natural scene with soil, branches, and leaves, is complex, relatively accurate segmentation results can be obtained. Because the strategy of the lesion image segmentation method used in this study was to determine the apple fruit location firstly and then determine the lesion location, the method required that the apple fruits should occupy the main part of the whole image in order to accurately determine the apple fruit location. In this study, it was assumed that the lesions of ring rot and anthracnose on apple fruits were nearly circular, but in practice, two or more lesions may join together, resulting in a great difference between the lesion shape and a circle, which could make the used lesion segmentation method ineffective. In addition, an apple fruit itself is a 3D (three-dimension) object, and a lesion on its surface is a 3D curved surface. The acquired image of the apple fruit and lesion is the projection of the 3D curved surface on a 2D (two-dimension) plane, and an inappropriate camera shooting angle can seriously affect the near circularity after the projection. Therefore, in the process of image capture, the lesions on the apple fruits should be photographed from the front side as much as possible to ensure the accuracy of lesion image segmentation. Meanwhile, the applications of 3D image acquisition and processing technology to plant disease distinction and identification should be strengthened.

Extracted features from the segmented lesion images are the bases of disease image recognition. However, uneven illumination and illumination changes can affect the extracted image features. In this study, the MB-LBP operators were used to extract the texture features in different scales and to reduce the influence of image noise caused by the factors including uneven illumination and illumination changes and based on the extracted features, satisfactory distinction results of the two apple diseases were achieved by using both the SVM method and the random forest method. As a local texture descriptor, the LBP operator has been utilized in plant disease image recognition (Leiva-Valenzuela and Aguilera, 2013; Dubey and Jalal, 2014, 2016; Shrivastava et al., 2017; Araujo and Peixoto, 2019). It has been improved to new operators including CLBP (Dubey and Jalal, 2014, 2016), adaptive center-symmetric local binary patterns (Wang et al., 2016), and square symmetric local binary patterns (Shrivastava et al., 2017) for image recognition of plant diseases. In further studies, more improved LBP operators can be used to explore better feature extraction methods for disease image recognition.

Deep learning has been applied to plant disease image recognition (Tan et al., 2015; DeChant et al., 2017; Lu et al., 2017; Liu et al., 2018; Bansal et al., 2021; Caldeira et al., 2021; Chen et al., 2021; Trivedi et al., 2021; Narmadha et al., 2022). It can reduce image preprocessing operations and achieve satisfactory disease recognition results. Compared with the disease distinction method used in this study, the deep learning method requires a large number of artificially labeled image samples to achieve satisfactory results; otherwise, the trained deep learning model is easy to result in over-fitting and poor generalization ability. In addition, the interpretability of a deep learning model is poor, and it is difficult to determine the reason for a recognition error made by using the model. In this study, the proposed segmentation methods for the images of apple ring rot and apple anthracnose were designed based on visual cognition experiences of human beings, and the purpose of each step involved in the segmentation algorithm is interpretable. When the number of the obtained image samples for training is not enough to meet the requirement of deep learning, the developed method in this study can provide a feasible solution. In further studies, it is expected to explore a general end-to-end apple disease recognition solution based on deep learning and a more comprehensive apple disease image database.

In recent years, image processing and recognition techniques have been developed and applied in many fields such as automatic apple picking (Zhang et al., 2016; Tao and Zhou, 2017; Kang et al., 2020), non-destructive detection of apple fruit quality (Zou et al., 2010; Zhang et al., 2014; Li Y. F. et al., 2021), automatic apples grading (Huang and Fei, 2017; Bhargava and Bansal, 2021), and apple yield estimation (Qian et al., 2013; Li Z. J. et al., 2021). These techniques can be used as references to carry out automatic identification and diagnosis of apple diseases. Computer vision technology can be made full use of to improve the ability of image acquisition and processing and to realize online detection and recognition of apple diseases. Furthermore, the distinction and identification of apple diseases could be implemented by comprehensive utilization of various detection methods. Hyperspectral imaging technology has been used to detect bruises (Xing et al., 2005; Ferrari et al., 2015; Tan et al., 2018; Zhang and Li, 2018) and insect damage (Tian et al., 2015; Rady et al., 2017) on apple fruits. The advantages of hyperspectral imaging technology with the characteristic of combining images with spectra can be used to detect apple diseases (Jarolmasjed et al., 2018; Shuaibu et al., 2018; Solovchenko et al., 2021).

The symptoms of plant diseases may be different at the different growth stages. The images with the typical symptoms of the two apple fruits diseases were used in this study. Further studies on the image distinction and recognition of the two apple fruit diseases with atypical symptoms are needed. Moreover, besides the two apple fruit diseases (ring rot and anthracnose), there are other apple fruit diseases such as scab, fruit moldy core rot caused by *Trichothecium roseum*, and Phytophthora rot caused by *Phytophthora cactorum* (Li B. H. et al., 2013; Hu et al., 2016). The research on image recognition technology for various apple fruit diseases and the different development stages of the diseases should be strengthened. Specially, the research on the early monitoring and early diagnosis of apple fruit diseases based on image processing technology should be carried out for effective and early control of the diseases. An image database of various apple fruit diseases should be established, and the image recognition methods and recognition systems for the diseases should be developed based on image processing technology. With the development and popularity of smartphones, image acquisition becomes more convenient. Apps (mobile applications) related to plant diseases have been developed and applied in practice. Smartphone-based apple disease recognition systems have been reported (Qu et al., 2015), but usually there are a few kinds of apple diseases included, leading to limited applicability. Therefore, the research and development of Apps for image recognition of various fruit tree diseases should be carried out so that the disease recognition could be conducted in the fields and the problems of the recognition and diagnosis of plant diseases could be solved in time. Furthermore, disease image distinction and identification can be integrated into an apple production management system or an apple post-harvest grading system to better ensure apple production safety and apple product management.

## Data Availability Statement

The original contributions presented in the study are included in the article/supplementary material, further inquiries can be directed to the corresponding author.

## Author Contributions

HGW contributed conception of the study. HGW and QF designed the experiments. QF, SW, HW, ZQ, and HGW performed the experiments. QF and HGW analyzed the data and wrote the draft of the manuscript. All authors contributed to manuscript revision, read, and approved the final version of the manuscript.

## Conflict of Interest

The authors declare that the research was conducted in the absence of any commercial or financial relationships that could be construed as a potential conflict of interest.

## Publisher’s Note

All claims expressed in this article are solely those of the authors and do not necessarily represent those of their affiliated organizations, or those of the publisher, the editors and the reviewers. Any product that may be evaluated in this article, or claim that may be made by its manufacturer, is not guaranteed or endorsed by the publisher.
